# Recent Progress in Thermoelectric Materials Based on Conjugated Polymers

**DOI:** 10.3390/polym11010107

**Published:** 2019-01-09

**Authors:** Chang-Jiang Yao, Hao-Li Zhang, Qichun Zhang

**Affiliations:** 1 School of Materials Science and Engineering, Nanyang Technological University (Singapore), Singapore 639798, Singapore; yaochemistry@iccas.ac.cn; 2 State Key Laboratory of Applied Organic Chemistry, Lanzhou University, Tianshui Southern Road 222, Lanzhou 730000, China

**Keywords:** thermoelectric, organic polymer, Seebeck coefficient, power factor, conductivity

## Abstract

Organic thermoelectric (TE) materials can directly convert heat to electricity, and they are emerging as new materials for energy harvesting and cooling technologies. The performance of TE materials mainly depends on the properties of materials, including the Seebeck coefficient, electrical conductivity, thermal conductivity, and thermal stability. Traditional TE materials are mostly based on low-bandgap inorganic compounds, such as bismuth chalcogenide, lead telluride, and tin selenide, while organic materials as promising TE materials are attracting more and more attention because of their intrinsic advantages, including cost-effectiveness, easy processing, low density, low thermal conductivity, and high flexibility. However, to meet the requirements of practical applications, the performance of organic TE materials needs much improvement. A variety of efforts have been made to enhance the performance of organic TE materials, including the modification of molecular structure, and chemical or electrochemical doping. In this review, we summarize recent progress in organic TE materials, and discuss the feasible strategies for enhancing the properties of organic TE materials for future energy-harvesting applications.

## 1. Introduction

About 90% of the world’s power is produced by heat engines that use fossil combustion as energy sources. However, fossil-based energy technologies cannot meet the fast-growing worldwide demand on electricity. Meanwhile, the development of new energy conversion technologies to address this issue is very urgent and highly desirable. Unfortunately, the maximum conversion efficiencies of most electricity-generation devices are limited at ca. 40% or lower, and a large amount of energy is lost as waste heat to the environment. Therefore, developing new technologies with improved energy conversion efficiency and reduced waste heat are urgent for addressing global environmental concerns, and they would provide new opportunities for the utilization of renewable energy resources.

**Figure 1:**
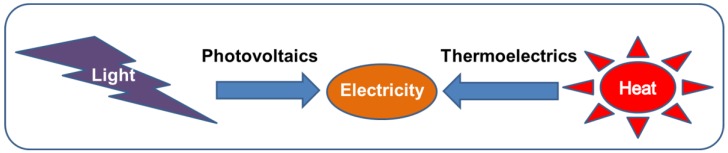
Electricity generated from thermoelectric and photovoltaics materials.

Thermoelectric (TE) and photovoltaics are two promising clean conversion technologies for solving the energy problem from an environmental–sustainable viewpoint (Figure 1). Compared to the great success in the development of photovoltaic materials in the last decade, the progress in the exploration of TE materials lags behind. TE materials utilize the temperature difference between the hot end and its surroundings to generate power [1], which can directly convert low-quality waste heat into the usable electric energy. Therefore, TE devices are promising candidates for making full use of waste heat and solar thermal energy [3].

**Figure 2:**
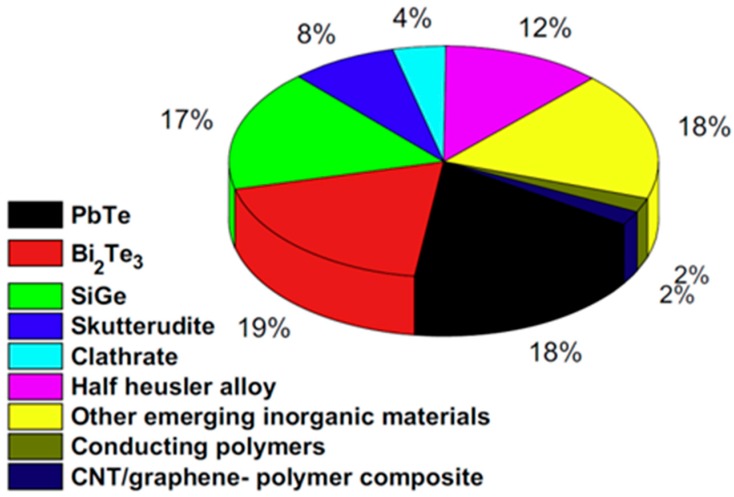
Schematic showing the various materials used in TE research, and their individual contributions. Reproduced with permission from [10].

**Figure 3:**
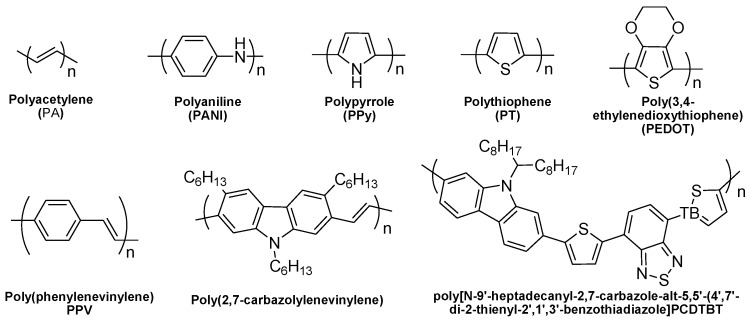
Chemical structures of representative p-type polymers for TE applications.

Conventional TE devices are typically based on inorganic compounds, as they generally show better TE performance and higher stability compared with organic materials. Some examples, including PbTe, Bi_2_Te_3_, SiGe, and Skutterudite, are widely explored, and the ZT value (a figure of merit) can reach as high as 2.6 [4,5,6] (Figure 2). However, inorganic semiconductor materials suffer some inherent disadvantages, including rarity, toxicity, poor processability, and a high cost of manufacturing. Besides, the ideal operation temperature for most high performance inorganic TE materials are generally above 200 degree, which cannot meet the increasing demand for collecting the waste heat generated at a temperature below 150 degree [7]. Organic semiconductors, which have been overlooked in the past decades due to their low energy conversion efficiency and possible poor thermal stability, however, may offer solutions to the above-mentioned problems. Organic semiconductors, such as conducting polymers, are promising candidates for converting low-end thermal energy into useful electricity [8], and they might enable the construction of sustainable, low-toxicity, larger-area, highly flexible thermoelectric devices [9]. In the last decade, great efforts have been input to improve the performance of organic TE materials, including the explosion of new molecular design strategy and the fabrication of nanocomposites consisting of conducting polymers and various nanomaterials. [10] The polymers applied in these researches are mainly conducting polymers (e.g., Poly(3,4-ethylenedioxythiophene (PEDOT), Polyaniline (PANI), and Polypyrrole (PPy)) (Figure 3).

## 2. Theory of TE Devices

The TE effect involves any transport phenomenon that implicates the relationship between heat and electrical potential energy. There are three reversible TE effects [11]: Seebeck for TE generation, Peltier effects for electronic refrigeration, and the Thomson effect. Although the non-zero TE effect might exist in all materials, the performance of a real TE device is lower than the Carnot efficiency, because of two irreversible processes: Joule heating and thermal conduction [12].

The performances of thermoelectric generators (TEGs) are mostly evaluated by the thermoelectric conversion efficiency *E* and the power output *P*. They are associated with the size of the TEGs, and TE materials and morphologies. The calculated thermoelectric conversion efficiency *E* is derived from thermodynamic theory (Equation (1)) [13,14,15]:

(1) $$E=\frac{\left(T_{1}-T_{2}\right)\cdot R_{L}}{T_{1}\left(R+R_{L}\right)-\frac{\left(T_{1}-T_{2}\right)\cdot R}{2}+\frac{{\left(R+R_{L}\right)}^{2}}{ZR}}$$

*T*_1_: Hot side temperature;
*T*_2_: Cold side temperature;
*k*: Thermal Conductivity;
*R_L_*: Load resistance;
*R*: Internal resistance of the semiconductor.

The Thomson effect is ignored for simplicity; thus, the Seebeck coefficient *S* is independent of temperature. The maximum conversion efficiency *E_max_* can be obtained when $R_{L}/R=\sqrt{\left(1+ZT\right)}$ (Equation (2)) and the maximum power output *P_max_* can be achieved under *R_L_* = *R* (Equation (3)).

(2) $$Emax=\left(T_{1}-T_{2}\right)\frac{\sqrt{1+Z\bar{T}}-1}{T_{1}\sqrt{1+Z\bar{T}}+T_{2}}$$

(3) $$Pmax=S^{2}{\left(T_{1}-T_{2}\right)}^{2}/\left(4R\right)$$

**Figure 4:**
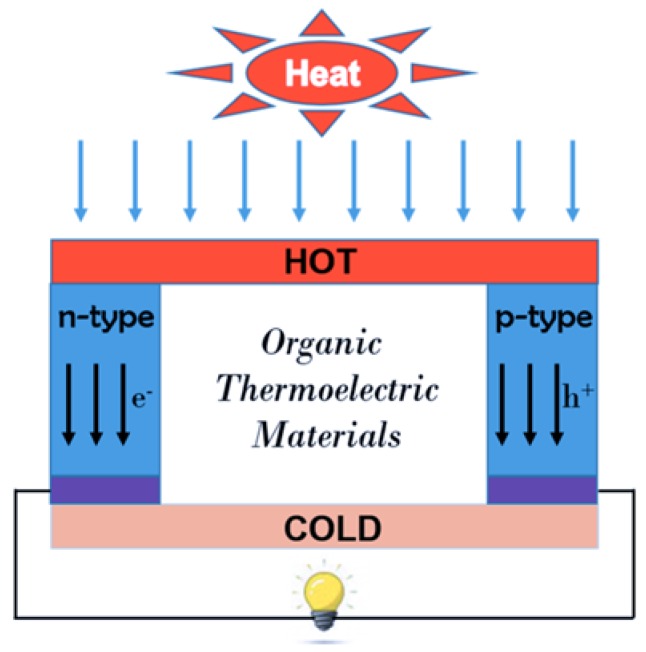
The illustration of the TE generator: Different temperature operated across the thermoelectric couple, and electrical power generated. Reproduced with permission from [16].

Since most materials show very small TE effects, and only a few semiconducting materials have potential in future practical applications. Also, to fulfil the function of the TE devices (Figure 4), both p-type and n-type semiconducting materials are simultaneously required in one TE device, and it is vital to choose the proper materials to match the generator voltage and current.

**Figure 5:**
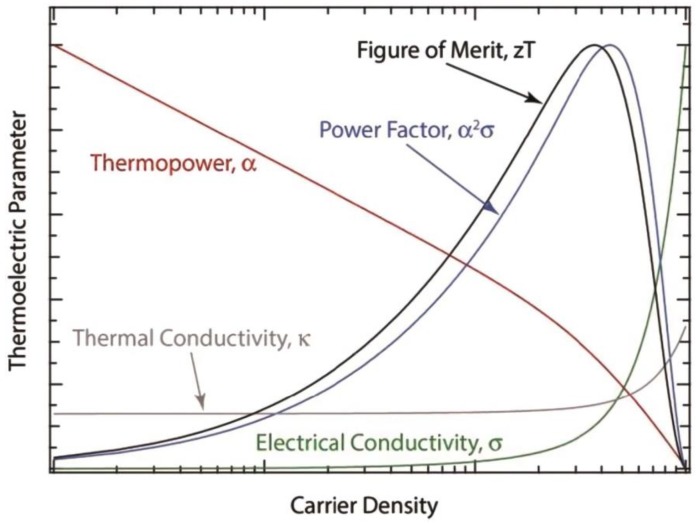
Schematic showing the coupled dependence of the various TE properties on carrier density. The shape of each individual curve was extracted from actual data on single-walled carbon nanotube networks. Figure reproduced with permission [19].

In early 1820, Thomas Johann Seebeck discovered the first TE effect in his experimental investigation on the relationship between electricity and heat [17]. A dimensionless TE figure of merit (*ZT*) is usually adopted to evaluate the efficiency of TE energy conversion. *ZT* is defined as *ZT* = *S*^2^*σT*/*k*, where *S* is the Seebeck coefficient, *σ* is the electrical conductivity (contributed from both phonons (or lattice conductivity) and electrons), *k* is the thermal conductivity, and *T* is the absolute temperature (Equation (4)). Hence, a higher TE performance can be achieved by larger *S*, higher *σ*, and lower *k*. Unfortunately, in most materials, these three variable parameters (*S*, *σ*, and *k*) are strongly interdependent, and materials with intrinsic high *ZT* are rare [18]. Recent investigation suggests that the engineering of micro-/nanostructures in composite materials could be an efficient way to address this issue, and to reach a higher *ZT* value (Figure 5), which could open new venues for the search and the design of TE materials, especially of composites consisting of organic semiconductors.

(4) $$ZT=\frac{S^{2}\sigma T}{k}$$

*S*: Seebeck Coefficient;
*σ*: Conductivity;
*T*: Temperature;
*k*: Thermal Conductivity.

The power generation efficiency at a determined temperature difference can be evaluated by the power factor (*PF* = *S*^2^*σ*). Thus, *ZT* is often replaced by *PF* to evaluate the thermoelectric performance of the organic polymer materials, and their related composites, due to their small thermal conductivities.

The reported theoretical studies on thermoelectric transport in polymers mainly aims to deal with the relationship between transport properties and the strength of the electron–phonon interaction [20]. Several kinds of models have been established based on polyamine in the 1990s, which mainly contributed to the explanation of the behavior of *S* and *σ* as a function of electric field, temperature, level of localization, etc. These models are further developed to study electric transport in disordered semiconductors. There are two typical models to evaluate the thermal resistance in materials: the diffuse mismatch model and the acoustic mismatch model [21]. Both are phenomenological approaches, assume linear phonon dispersions, oversimplify the effects from the interfaces, and are fitted to reproduce existing experimental results.

Except for these simplified approaches, molecular dynamic (MD) techniques are also applied to analyze the thermal conductivity of polymers, based on classical mechanics [22]. The ab initio is an alternative method to calculate the thermal properties under the using the Onsager relations, and the Landauer theory of quantum transport [23], and the thermodynamics theory establishes linear relations between conjugate “flows” and “driving forces” through proportionality coefficients [24]. The electrical and heat currents both generate concomitantly in a thermoelectric system, and they interact each other, and the two forces can generate either of two flows or both of them at the same time [25].

## 3. p-Type TE-Conducting Polymers

The most typical organic materials bearing TE properties are conducting polymers (e.g., PEDOT, PANI and PPy) (Figure 3), which possess many charming features, including high electrical conductivity, low bad gap energy, environmental and thermal stability, a light weight and strong backbone, and easy processability.

As one leg of the TE devices, p-type TE organic semiconductors (OSCs) [26,27,28,29] have been extensively studied. For example, PEDOT derivatives can achieve the highest power factor *PF* over 300 μW·m^−1^ with *ZT* around 0.25 [8]. The TE performance of typical p-type traditional conducting polymers such as polyaniline (PANI), polypyrrole (PPY) [30,31], polythiophene (PTH), poly(3,4-ethylenedioxythiophene): poly(styrenesulfonate)/tosylate (PEDOT:PSS, PEDOT-Tos), polyacetylene (PA) [32], polycarbazoles (PC) [33], polyphenylenevinylene (PPV), and their derivatives have been widely studied as potential TE materials, and their performances are summarized in Table 1. The intrinsic conductivities of most conducting polymers are in the range of 10^−16^–10^−5^ S/cm, while their intrinsically thermal conductivities typically lie between 0.11 and 0.4 W/mK, which is beneficial for *ZT* value [34]. Currently, the power factors (*PF*) for most polymer-based TE materials are in the range of 10^−6^–10^−10^ Wm^−1^ K^−2^, which is much smaller compared with that of conventional inorganic TE materials [35,36,37,38]. However, the insulating behaviors of the organic materials in the pristine state can be depressed after doping. In the following sections, we will discuss the performance of some of p-type conjugated polymers in detail, in order to understand the relationship between the structures and TE performance.

### 3.1. Polyacetylene (PA)

The iodine-vapor-doped polyacetylene has electrical conductivity as high as 10,000 S·cm^−1^ [61,62]. Employing doped polyacetylene films as active elements for TE measurement, Na et al. found that the variation of temperature can influence the electrical conductivity, and the maximum conductivity can achieve 30,000 S·cm^−1^ at *T* = 220 K in the FeCl_3_-doped stretchable polyacetylene films [63]. Later on, the polyacetylene films doped with iodine and FeCl_3_ have been demonstrated to show *PF* of up to 2 × 10^−3^ W·m^−1^ K^−2^ [47] and 8.3 × 10^−5^ W·m^−1^ K^−2^ [64], respectively. However, the further development of polyacetylene as a TE material is limited by its insolubility and poor air stability.

### 3.2. Polyaniline (PANI)

Since PANI has many attractive properties, such as high conductivity, good stability, and easy preparation/processing, it has become the most commonly studied TE material among all conducting polymers [65]. The electrical conductivity of (±)-10-camphorsulfonic acid (CSA)-doped PANI can achieve 300 S/m, and the Seebeck coefficient reaches 8–12 µV/K at 300 K. It is noted that a PANI-based multilayered film doped with CAS shows a six-fold increase in ZT, compared to that of bulk CSA-doped PANI, and the ZT value of the former one can reach 1.1 × 10^−2^ at 423 K. One also should notice that the dopant agents can greatly affect the performance of TE polymers; for example, the maximum *ZT* value of HCl-doped PANI can only give 2.67 × 10^−4^ at 423 K [66,67].

The excellent thermal stability and low thermal conductivity of PANI are attractive for its application in TE devices; however, its electrical conductivity is greatly associated with the morphology, molecular weight, chain arrangement, oxidation level, and doping level. Thus, further enhancing the electrical conductivity can be realized through optimizing these parameters. In addition, the cheap starting materials can be one advantage for making PANI film more promising for practical applications if its *ZT* value can be further improved by dopants or other methods.

### 3.3. Polypyrrole

Although polypyrrole (PPy) has the merits of common conducting polymers, such as relatively easy processing, low cost, and good environment stability, there are less reports on their applications, as TE materials, compared with their usage in electronic energy devices. The major efforts lie on PPy-based nanocomposites. Since carbon nanotubes (CNTs) are quite powerful thermoelectric materials as well, they have been widely used to blend with the polymers to improve the TE performance. Polymer–CNT nanocomposites can be prepared by many methods, such as in situ polymerization, dissolving polymers into organic solvents containing multiwall carbon nanotube (MWNT) suspensions, melt mixing, grafting macromolecules onto CNT, and interfacial polymerization. Zhang et al. studied the performance of PPy-nanotube films with varied sizes of CNTs [68]. In their research, they fabricated two flexible and freestanding polypyrrole (PPy) nanotube films (PPy-1 and PPy-2), and carefully studied their structures, morphologies, and thermoelectric properties. The PPy film with shorter PPy nanotubes gave a Seebeck coefficient of 16.5 μV·K^−1^, electrical conductivity of 3.43 S·cm^−1^, and *PF* of 0.09 μW·m^−1^·K^−2^, while the longer PPy nanotubes showed an electrical conductivity, Seebeck coefficient, *PF* at 9.81 S·cm^−1^, 17.68 μV·K^−1^, 0.31 μW·m^−1^·K^−2^, respectively. These results indicate that the longer length and smaller sizes of the PPy nanotubes were helpful for enhancing the electrical conductivity and the Seebeck coefficient, while size and length have less effects on thermal conductivity.

The nanoscale structures of the PPy-based materials also affect their TE properties. Guo et al. found that, among the different morphologies of the as-prepared PPy nanostructures such as nanoparticles and nanowires, and the nanowires with largest aspect ratios exhibited the highest *PF* of (22.6 ± 3.6) × 10^−3^ μW·m^−1^·K^−2^ [69]. This result suggests that the development of novel organic thermoelectric materials can be achieved by morphology-controlling strategy.

### 3.4. Polythiophene-Based Derivatives

Generally, polythiophene (PT) has a low Seebeck coefficient and a low electrical conductivity, and its TE performance is strongly affected by the sizes of the side chains and the main chain structure. For example, the electropolymerized freestanding polythiophene and poly(3-methylthiophene) (PMeT) nanofilms can exhibit high Seebeck coefficients (130 and 76 µV/K), decent electrical conductivities (47 and 73 S/cm), and low thermal conductivities (0.17 and 0.15 W/mK) at room temperature, compared with those made by other method [39]. Due to the good rearrangement of polymer chains in the electropolymerized films, PT and PMeT could display much better TE performances than those obtained in pressed pellet samples.

The polyethylenedioxythiophene (PEDOT) family is the most popular TE polymers because of their excellent electrical conductivity, power factor, and its high stability toward moisture and oxygen. The reported research have already indicated that TE devices based on PEDOT can have very good performances [58,70,71,72]. The first PEDOT-based TE device was reported in 1988 [73], and its Seebeck coefficient and electrical conductivity can be optimized by controlling the dopant concentration and the oxidation level during the polymerization.

Normally, there are two common methods to fabricate conductive PEDOT films. One is in situ polymerization, namely, the production of PEDOT during film formation. For example, the PEDOT:tos (tos: tosylate) film can be in situ prepared by using Fe(tos)_3_ as an oxidizing agent, and the highest electrical conductivity of the as-prepared thin film can reach 4300 S/cm [59,74]. The other method is to use a stable PEDOT dispersion solution to cast a film. However, this method is only limited to the PEDOT:PSS system. PEDOT dispersion solution is cheap enough to fabricate large-area devices, and its doped state can also be easily processed through solution-processing techniques.

The TE performance of PEDOT/PSS(poly(4-styrenesulfonate)) has been extensively studied by many groups [71]. The effects of dielectric solvents (e.g., DMSO) and the ratio between PEDOT and PSS in the polymeric films were carefully investigated [59]. The authors found that the Seebeck coefficient can keep unchanged within certain ranges of DMSO and PSS concentrations, while the electrical conductivity becomes the major factor for controlling the value of *PF*.

**Figure 6:**
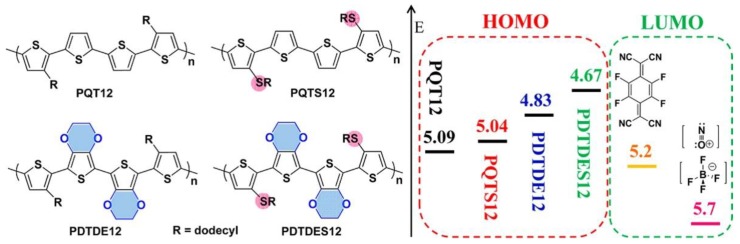
Chemical structures of polymers and dopants and their Highest Occupied Molecular Orbital (HOMO) and Lowest Unoccupied Molecular Orbital (LUMO) energy levels, respectively. Reproduced with permission from [75].

Katz et al. modified the structure of p-type poly(bisdodecylquaterthiophene) (PQT12) (Figure 6) to realize high and predominantly nonionic conductivity. [75] The highest conductivities of up to 350 S·cm^−1^ and 140 S·cm^−1^ were achieved for NOBF_4_-doped PQTS12 and F_4_TCNQ-doped PDTDE_12_, respectively. Tighter π–π stacking, efficient charge transfer, and strong intermolecular coupling have major contribution to the conductivity. The conductivities of these polymers are stable in air, without extrinsic ion contributions. These results may suggest that further improving the ZT value could be realized by increasing the mobilities of the polymer through doping.

### 3.5. Other p-Type TE Polymers

Poly(2,7-carbazole) (PC) and their derivatives were also investigated as p-type TE materials [76,77]. Although polycarbazole derivatives usually show high Seebeck coefficients; however, their electrical conductivities are small [57]. The conductivity of polycarbazole (PC) derivatives can be significantly enhanced by the introduction of vinylene and electron-donating groups such as thiophene or bis(3,4-ethylenedioxythiophene) [37]. A Seebeck coefficient of 34 μV·K^−1^, an electroconductivity of 160 S·cm^−1^, and a corresponding *PF* of 19 μW m^−1^·K^−2^ were obtained for a polycarbazole derivative, poly[*N*-9′-heptadecanyl-2,7-carbazole-alt-5,5′-(4′,7′-di-2-thienyl-2′,1′,3′-benzothiadiazole] (PCDTBT) (Figure 3) [57].

Some other conducting polymers such as polyparaphenylene, polyparaphenylene vinylene, and poly(2,5-dimethoxphenylenevinylene) (PMeOPV), and a series of copolymers have also been investigated as TE material [39]. However, their performance needs further improvement.

## 4. TE Properties of n-Type Polymers

**Figure 7:**
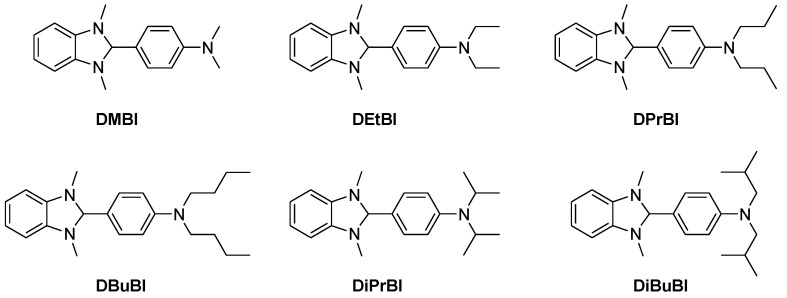
Molecular structures of well-known n-dopants: *N*-alkyl-substituted 1H-benzimidazoles.

Compared to p-type polymer TE materials, their n-type counterparts exhibit much poorer TE behaviors, due to inefficient doping and much lower conductivity. Also, the preparation of n-type polymer TE materials is also hindered by their air-sensitive character, which makes their research lag far behind. It is well-known that the stability of the n-type polymers is strongly related to the LUMO level, and the deeper LUMO level of the n-type polymers is a prerequisite for ensuring the realization of high conductivity [78] (Table 2). Highly stable n-type thermoelectric materials can be realized by mixing carbon nanotubes with conjugated polymers, and these novel n-type thermoelectric composites possess longer operation lifetimes and air stabilities. Typically, *N*-alkyl substituted 1H-benzimidazoles are employed as n-dopants for organic thermoelectric materials (Figure 7).

### 4.1. Building Units for n-Type Polymers

**Figure 8:**
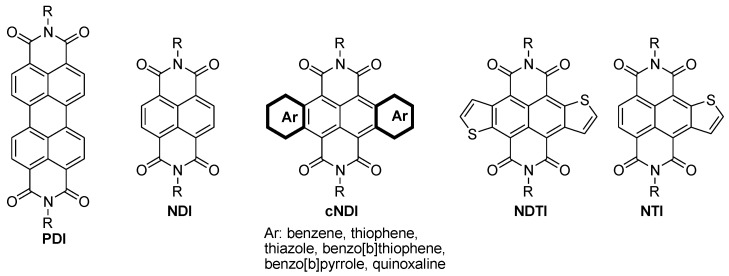
The building blocks for n-type polymers.

Perylene bisimide [90], naphthalenetetracarboxylic dianhydride [91], naphthodithiophenediimide [92], benzotriazole [93], and fullerenes [79] have been widely used as n-type organic small molecules for organic semiconductor devices (Figure 8). The LUMO level of polymers based on these building units can be adjusted by introducing different electron-donating units into these polymer structures.

Naphtho[2,3-b:6,7-b′]dithiophenediimide (NDTI) and naphtha[2,3-b]thiophene diimide (NTI) featured with low-lying energy levels of LUMO (3.8–4.1 eV) and the easy functionalizability of the α-positions in thiophene, which allow the derivatives and polymers to conjugate efficiently with additional π- and comonomer units. Recently, various air-stable doped n-type semiconductors derived from the NDTI and NTI are applicable to n-type TE materials with high electron mobility (~0.8 cm^2^·V^−1^·s^−1^) [94,95].

**Figure 9:**
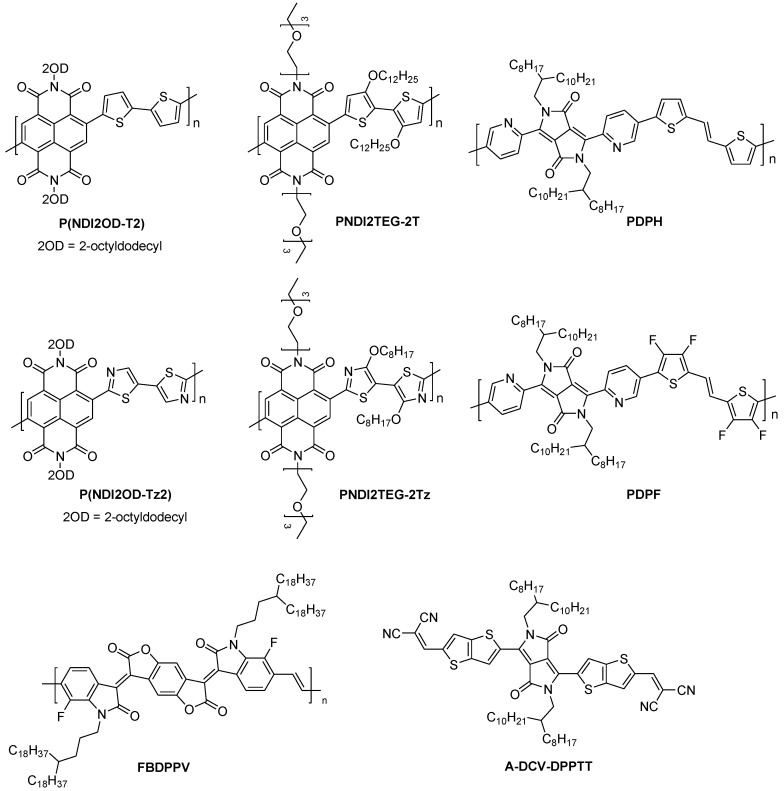
Chemical structures of some representative n-type polymers.

Facchetti et al. investigated the morphological, structural, charge transport, and thermoelectric properties of the films, based on naphthalenediimide (NDI)-bithiazole (Tz2)-based polymer [P(NDI2OD-Tz2)] [96] (Figure 9). They found that P(NDI2OD-Tz2) possess a more planar and rigid backbone, and exhibited enhancing π–π stacking, as well as intermolecular interactions, compared with the widely-investigated parent polymer P(NDI2OD-T2). Furthermore, the electron affinity of the as-prepared polymer was improved, due to the electron-deficient nature of Tz2, thus decreasing the polymer donor–acceptor character. Decent electrical conductivity (≈0.1 S·cm^−1^) and a reasonable power factor (1.5 µW m^−1^·K^−2^) were achieved for P(NDI2OD-Tz2) after amine doping. These data are higher than those of the undoped P(NDI2OD-T2) (0.003 S·cm^−1^ and 0.012 µW m^−1^·K^−2^, respectively). These results suggest that reducing donor–acceptor character of planarized NDI-based polymers can lead to substantial electrical conductivity and thermoelectric response.

The chemical structure of the polymers, and the solid-state packing of interacting conjugated segments can largely affect charge transport in polymer films [97]. Bertarelli et al. have studied how the alkyl substitution on the backbones of air-s H-benzimidazole-based dopants, to affect the morphology and electrical properties of the copolymer P(NDI2OD-T2) [98] after blending (Table 1). It turns out that higher *σ* values can be obtained from the dopants with longer linear alkyl chains. For example, *N*-butyl-substituted dopants can reach four times higher conductivities (4.1 × 10^−3^ S·cm^−1^) than these in the methyl-substituted reference ones. Compared to pristine P(NDI2OD-T2) films, the structural analysis suggests that an edge-on orientation of polymer crystallites, a decrease in the lamellar stacking distance, and a disruption of backbone and π-stacking order were induced by the dopant molecules. The introduction of the soluble chains in small molecule-based dopants is an effective method for controlling and enhance the miscibility of dopants and polymers. This method allows to improve the conductivity of polymers due to the improved doping efficiency.

Till now, there are two different proposed models to describe the molecular doping of organic semiconductors: the molecular orbital hybridization (MOH) model and the inter-charge transfer (ICT) model [99]. Koster et al. found that power factor of n-type organic thermoelectric donor–acceptor (D–A) copolymers can be increased by a factor of > 1000 through tailoring of the density of states (DOS) [100,101]. The DOS distribution of PNDI2TEG-2T is tailored by the embedded sp^2^-nitrogen atoms at a donor unit of the D-A copolymer (Figure 9). Consequently, PNDI2TEG-2Tz achieved an electrical conductivity of 1.8 S·cm^−1^, and a power factor of 4.5 µW·m^−1^ K^−2^.

Pei and Zhu et al. reported that the n-doping efficiency of a D–A polymer can be enhanced by the introduction of electron-deficiency F atoms into donor units of the polymer backbone (PDPF). A high n-type electrical conductivity of 1.30 S·cm^−1^ and an excellent power factor (*PF*) of 4.65 µW·m^−1^K^−2^ were obtained, which were enhanced for three orders of magnitude, comparing to the non-engineered polymer (PDPH) [96]. They suggested that there are three advantages of the electron-withdrawing modification of the donor moiety in a D–A conjugated polymer: (1) intramolecular H-bonds are formed, and both the HOMO and LUMO levels of the polymer are lowered; (2) the improvement of the n-doping efficiency and the avoid of phase separation; and (3) the lowering of the carrier hopping barrier while the mobility remains unaffected, or even greatly enhanced.

**Figure 10:**
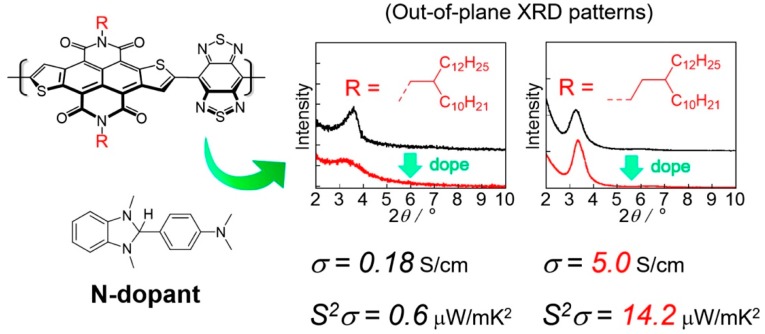
Naphthodithiophenediimide-benzobisthiadiazole-based polymers. Reprinted with permission from [102].

Nakano and Takimiya et al. have developed several new π-conjugated polymers PNDTI-BBTs (Figure 10) with strong electron affinity, including naphtho[2–b:6,7-b′]dithiophenediimide (NDTI) and benzo[1,2-c:4,5-c′]-bis[1,2,5]thiadiazole (BBT) units [102]. The low-lying LUMO energy levels (~−4.4 eV) of PNDTI-BBTs make them readily doped by *N*,*N*-dimethyl-2-phenyl-2,3-dihydro-1H-benzoimidazole (N-DMBI), providing the doped polymer films, with relatively high Seebeck coefficients and conductivities. The conductivity and the power factor of the doped thin films (~0.18 S·cm^−1^ and ~0.6 μW·m^−1^·K^−2^ for PNDTI-BBT-DT; ~5.0 S·cm^−1^ and ~14 μW·m^−1^·K^−2^ for PNDTI-BBTDP) were dramatically changed by the usage of soluble alkyl groups such as 2-decyltetradecyl, PNDTI-BBT-DT, or 3-decylpentadecyl groups, and PNDTI-BBT-DP. The better crystalline nature of PNDTI-BBT-DP than PNDTI-BBT-DT in the thin film was the main reason for causing the differences in their electric properties.

**Figure 11:**
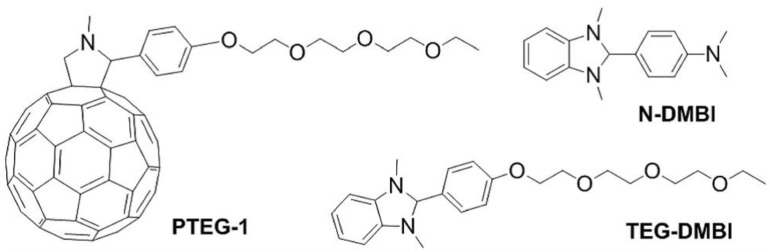
Chemical structures of PTEG-1, N-DMBI, and TEG-DMBI. Reprinted with permission from [103].

Hummelen et al. reported a promising n-type doping system [103], which is constructed by introducing polar triethylene glycol (TEG) side chain onto both fullerene host (PTEG-1) and dopant (TEG-DMBI) materials (Figure 11). The improved miscibility of solution-processed TEG-DMBI-doped PTEG-1 films displayed a higher carrier density and mobility compared with N-DMBI-doped films. A record power factor of 19.1 mW·m^−1^·K^−2^ and an electrical conductivity of 1.81 S·cm^−1^ were achieved.

### 4.2. Poly(nickel-ethylenetetrathiolate) (KxNiett)

Since Poleschner et al. firstly reported the poly(Ax(M-ett)) system 30 years ago, the synthesis, electrical/magnetic properties, and the structure of poly(Ax(M-ett)) system have been studied by many groups. Although these polymers have excellent conductive properties (~10^−5^–50 S·cm^−1^), there are few investigations on their TE performances. Recent research progress in the thermoelectric properties of poly(M-ett) (M = metal, ett = ethylenetetrathiolate) showed that poly(M-ett) provided the best performance of n-type organic thermoelectric materials.

Yang et al. reported that the intrinsic metallic behaviors and high-performance TE power factors can coexist within dopant-free linear-backbone conducting polymers, poly(nickel-ethylenetetrathiolate) and its analogs. Based on density functional calculations, Yang and Xue et al. found that four crystalline π-*d* conjugated transition–metal coordination polymers such as poly(Pd-C_2_S_4_), poly(Pt-C_2_S_4_), poly(Ni-C_2_S_4_), and poly(Ni-C_2_Se_4_) showed intrinsic metallic behaviors, generating from dense intermolecular interaction networks between sulfur/selenium atoms. Moderate carrier concentrations (10^19^–10^21^·cm^−3^) and decent conductivities (10^3^–10^4^ S·cm^−1^) have been found in these polymers. Especially, poly(Ni-C_2_S_4_), poly(Ni-C_2_Se_4_), and poly(Pd-C_2_S_4_) exhibit high power factors (~10^3^ μW·m^−1^·K^−2^) [104].

## 5. Strategies for Enhancing the ZT of Organic TE Materials

According to the definition of the figure of merit, *ZT* = *S*^2^*σT*/*k*. A desired TE material should have the following properties: a large Seebeck coefficient to promote the energy conversion of heat to electricity, a high electrical conductivity to reduce Joule heating, and a low thermal conductivity to prevent thermal shorting. All of the current research is focused on these aspects through the following strategies.

### 5.1. Molecular Structure Modification

An effective TE material should have an enhanced Seebeck coefficient and a high electrical conductivity simultaneously, while remaining small *κ* constant. Chemical functionalization is an effective way towards improving *ZT* through increasing the carrier mobility and keeping a constant carrier density, which leads to an improved Seebeck coefficient and an enhanced electrical conductivity simultaneously, according to the equation *σ* = *enµ*, where *e* is the electron charge, *n* is the charge carrier density, and *µ* is the carrier mobility.

The theoretical calculation based on the existing data can conclude some useful structure–property relationships, which can instruct scientists to design efficient TE materials for real applications.

### 5.2. Nanocomposites of Organic/Inorganic Hybridization

Compared to conventional rigid inorganic thermoelectric (e.g., PbTe or Bi_2_Te_3_), organic thermoelectric shows obvious advantages for their easy fabrication into versatile formats. However, their performance is limited by their inferior electrical conductivity, a small Seebeck coefficient, and a low power factor (*PF*). This issue might be solved by nanocomposited organic/inorganic hybrid films, because these hybrid films can integrate low thermal conductivities of organic TE materials and high electroconductivity/Seebeck values of inorganic counterparts together, leading to higher TE materials, such as HH alloys, skutterudites, and Si–Ge systems. Polymer/inorganic TE nanocomposites can be prepared by solution mixing, physical mixing, interfacial polymerization, or in situ chemical oxidative polymerization. Several nanocomposites, including PANI/NaFe_4_P_12_, PANI/PbTe, PEDOT/Na_2_TeO_3_, and PEDOT/Ca_3_Co_4_O_9_ can be fabricated by in situ chemical oxidative polymerization, while PANI/BiTe_3_ and PEDOT/Bi_2_Te_3_ composites can only be realized by a physical mixing method, due to the easy oxidation of Bi_2_Te_3_ during the process of in situ chemical oxidative polymerization [105]. Carbon nanotubes (CNTs) are one-dimensional carbon nanomaterials with nanoscale diameters and single or multiple walls, and they can be employed as effective conductive fillers in polymer films for achieving high ZTs, due to their high electrical conductivity, excellent mechanical properties, and high thermostability. [106] Generally, the organic/inorganic composites can exhibit a higher Seebeck coefficient, compared to those of pure polymers [107].

### 5.3. Organic Polymer Doped with Fillers

The high electrical conductivity of the conductive polymers could be realized by the doping method, however, their Seebeck coefficient is compromised, leading to very low *ZT*. In order to address this issue, conjugated polymer-based nanocomposites might be a good solution, because the electrical conductivities of these materials was dramatically enhanced, while their thermal conductivity and Seebeck coefficient remain insensitive to the filler concentration. Note that the fillers of TE composites can be either inorganic or organic, while the matrix can be either insulating or intrinsically conductive.

## 6. Conclusions and Perspectives

In this review, we summarize recent progresses in organic thermoelectric materials. In contrast to conventional rigid inorganic thermoelectrics, flexible organic thermoelectrics show obvious advantages for their easy fabrication into versatile formats. However, organic materials still suffer from poor thermoelectric properties. Thus, developing new strategies to enhance the TE performance of conjugated polymers will still be the future focus.

The deeply understanding of the structure–property relationship is very important for the exploration of novel organic thermoelectric materials. The theoretical modelling of the relationships between molecular structures, energy levels, density of state, and thermoelectric performance can provide instructive indication for the further development. Since the thermoelectric performance (*ZT* value) greatly relies on the interdependence of *S*, *σ*, and *k*. The theoretical prediction work is expected to further advances in experimental activities. Also, the further understanding on the relationship between the molecular structure and property needs to be studied through the synthesis of more polymers in future. In addition, post-treatment approaches to optimize the morphology and the doping level of different dopant-doped polymers should also be developed.

Apart from the classical conducting polymers such as PANI, PEDOT, and PPy, which have been broadly studied as p-type TE materials, some n-type organic TE materials based on PDI, NDI, NDTI, and NTI units have been constructed, and their TE performance have been summarized. The complete efficient TE generators compose of both p-type and n-type materials. However, the n-type organic TE materials with comparable performance are still rare, due to their poor air stability. Thus, more efficient n-type organic TE materials are greatly in demand.

To make full use of the intrinsic advantages of organic thermoelectric and extend TE applications under different environments and on various surfaces, flexible and stretchable devices of polymer composites are strongly suggested. To further realize industrial TE applications, an effective way to improve the *ZT* of organic polymer materials is highly desirable. In fact, organic/inorganic hybrid films, which integrate mechanical flexibility, stretchability, and solution processing from the organic component and higher thermoelectric properties from the inorganic part together, would be a promising direction to achieve higher *ZT*.

## Figures and Tables

**Table 1:** The *S*, *σ*, *k* and *ZT*_max_ values of a few typical p-type polymers [39].

Polymer	*S* μV/K	*σ* S/cm	*k* W/mK	*ZT*_max_	*PF*_max_ μW/m K^2^	Ref.
**PANI**	−16–225	10^−7^–320	0.02–0.542	1.1 × 10^−2^ at 423 K		[40,41,42,43,44,45]
**PA**	−0.5–1077	1.53 × 10^−3^–2.85 × 10^4^				[46,47,48,49,50,51]
**PT**	10–100	10^−2^–10^3^	0.028–0.17	2.9 × 10^−2^ at 250 K		[52,53,54,55]
**PPy**	−1–40	0–340	0.2	3 × 10^−2^ at 423 K		[31,56]
**PC**	4.9–600	4.0 × 10^−5^–5 × 10^−2^			19	[33,57]
**PEDOT:PSS**	8–888	0.06–945	0.34	1.0 × 10^−2^ at 300 K		[43,44,58,59,60]
**PEDOT-Tos**	40–780	6 × 10^−2^–300	0.37	0.25 at RT		[8]

**Table 2:** Summary of the TE performance of typical n-type polymers [78].

Polymer	Dopant	*S* [μV·K^−1^]	*σ* [S·cm^−1^]	*k* [W·m^−1^·K^−1^]	*PF* [μW·m^−1^·K^−2^]	*ZT*	Ref
**PA**	Bu_4_N	−43.5	5	-	1	-	[61]
**C_60_**	Cr_2_(hpp)_4_	−175	4		12		[79]
**FBDPPV (Figure 9)**	DMBI	−141	14	-	28	-	[80]
**A-DCV-DPPTT (Figure 9)**					105	0.11	[81]
**FBDPPV (Figure 9)**	DMBI	−210	6.2	-	25.5	-	[82]
**P(NDIOD-T_2_)**	N-DMBI	−850	0.008	-	0.6	-	[83]
**P(NDIOD-T_2_)**	N-DPBI	−770	0.004	-	0.2	-	[83]
**K_x_C_70_**		−22.5	550		28		[84]
**BBL**	TDAE	−101	0.42	-	0.43	-	[85]
**Poly[K_x_Ni-ett]**	N. A	−122	40	−0.2	66	−0.1	[86]
**KxNi-ett**	N. A	−90	210	0.4–0.5	170	0.30	[87]
**CuBHT**	N. A	−4 to −10	750–1580	-	N. A.	-	[88]
**P_3_HT**	Doped with PF_6_	39	0.843		0.14		[89]

## References

[1] Goldsmid H.J., Douglas R.W.D. (1954). The use of semiconductors in thermoelectric refrigeration. Br. J. Appl. Phys..

[2] Sun Y., Xu W., Di C.A., Zhu D. (2017). Metal-organic complexes-towards promising organic thermoelectric materials. Synth. Met..

[3] Snyder G.J., Toberer E.S. (2008). Complex thermoelectric materials. Materials for Sustainable Energy.

[4] Zhao L.D., Lo S.H., Zhang Y., Sun H., Tan G., Uher C., Wolverton C., Dravid V.P., Kanatzidis M.G. (2014). Ultralow thermal conductivity and high thermoelectric figure of merit in SnSe crystals. Nature.

[5] Venkatasubramanian R., Silvola E., Colpitts T., O’quinn B. (2001). Thin-film thermoelectric devices with high room-temperature figures of merit. Nature.

[6] Pei Y., Shi X., LaLonde A., Wang H., Chen L., Snyder G.J. (2011). Convergence of electronic bands for high performance bulk thermoelectrics. Nature.

[7] Wei Q., Mukaida M., Kirihara K., Naitoh Y., Ishida T. (2015). Recent Progress on PEDOT-Based Thermoelectric Materials. Materials.

[8] Bubnova O., Khan Z.U., Malti A., Braun S., Fahlman M., Berggren M., Crispin X. (2011). Optimization of the thermoelectric figure of merit in the conducting polymer poly(3,4-ethylenedioxythiophene). Nat. Mater..

[9] Asano H., Sakura N., Oshima K., Shiraishi Y., Toshima N. (2016). Development of ethenetetrathiolate hybrid thermoelectric materials consisting of cellulose acetate and semiconductor nanomaterials. Jpn. J. Appl. Phys..

[10] Gayner C., Kar K.K. (2016). Recent advances in thermoelectric materials. Prog. Mater. Sci..

[11] Bubnova O. (2013). Thermoelectric Properties of Conducting Polymer. Ph.D. Thesis.

[12] Sherman B., Heikes R.R., Ure R.W. (1960). Calculation of Efficiency of Thermoelectric Devices. J. Appl. Phys..

[13] Ma Q., Fang H., Zhang M. (2017). Theoretical analysis and design optimization of thermoelectric generator. Appl. Therm. Eng..

[14] Chen J., Yan Z., Wu L. (1996). The influence of Thomson effect on the maximum power output and maximum efficiency of a thermoelectric generator. J. Appl. Phys..

[15] Ruciński A., Rusowicz A. (2017). Thermoelectric Generation of Current—Theoretical and Experimental Analysis. Arch. Thermodyn..

[16] Fleurial J.-P. (2009). Thermoelectric Power generation materials: Technology and application opportunities. JOM.

[17] Seebeck T.J. (1822). Magnetic polarization of metals and minerals. Abh. Dtsch. Akad. Wiss. Berl..

[18] Dresselhaus M.S., Chen G., Tang M.Y., Yang R., Lee H., Wang D., Ren Z., Fleurial J.-P., Gogna P. (2007). New Directions for Low-Dimensional Thermoelectric Materials. Adv. Mater..

[19] Blackburn J.L., Ferguson A.J., Cho C., Grunlan J.C. (2018). Carbon-Nanotube-Based Thermoelectric Materials and Devices. Adv. Mater..

[20] Chen X.P., Jiang J.K., Liang Q.H., Yang N., Ye H.Y., Cai M., Shen L., Yang D.G., Ren T.L. (2015). First-principles study of the effect of functional groups on polyaniline backbone. Sci. Rep..

[21] Mao R., Kong B.D., Kim K.W., Jayasekera T., Calzolari A., Buongiorno Nardelli M. (2012). Phonon engineering in nanostructures: Controlling interfacial thermal resistance in multilayer-graphene/dielectric heterojunctions. Appl. Phys. Lett..

[22] Landry E.S., McGaughey A.J. (2009). Thermal boundary resistance predictions from molecular dynamics simulations and theoretical calculations. Phys. Rev. B.

[23] Peng S., Wang D., Lu J., He M., Xu C., Li Y., Zhu S. (2017). A Review on Organic Polymer-Based Thermoelectric Materials. J. Polym. Environ..

[24] Onsager L. (1931). Reciprocal relations in irreversible processes. I. Phys. Rev. B.

[25] Arnas O.A., Miller D.L. On an irreversible thermodynamic analysis of thermoelectric devices. Proceedings of the 2nd International Conference on Thermoelectric Energy Conversion.

[26] Glaudell A.M., Cochran J.E., Patel S.N., Chabinyc M.L. (2015). Impact of the Doping Method on Conductivity and Thermopower in Semiconducting Polythiophenes. Adv. Energy Mater..

[27] Russ B., Glaudell A., Urban J.J., Chabinyc M.L., Segalman R.A. (2016). Organic thermoelectric materials for energy harvesting and temperature control. Nat. Rev. Mater..

[28] Zhang Q., Sun Y., Xu W., Zhu D. (2014). Organic Thermoelectric Materials: Emerging Green Energy Materials Converting Heat to Electricity Directly and Efficiently. Adv. Mater..

[29] Patel S.N., Glaudell A.M., Peterson K.A., Thomas E.M., O’Hara K.A., Lim E., Chabinyc M.L. (2017). Morphology controls the thermoelectric power factor of a doped semiconducting polymer. Sci. Adv..

[30] Mateeva N., Niculescu H., Schlenoff J., Testardi L.R. (1998). Correlation of Seebeck coefficient and electric conductivity in polyaniline and polypyrrole. J. Appl. Phys..

[31] Kemp N.T., Kaiser A.B., Liu C.J., Chapman B., Mercier O., Carr A.M., Trodahl H.J., Buckley R.G., Partridge A.C., Lee J.Y. (1999). Thermoelectric Power and Conductivity of Different Types of Polypyrrole. J. Polym. Sci. Part B Polym. Phys..

[32] Park Y.W., Han W.K., Choi C.H., Shirakawa H. (1984). Metallic nature of heavily doped polyacetylene derivatives: Thermopower. Phys. Rev. B.

[33] Lévesque I., Bertrand P.O., Blouin N., Leclerc M., Zecchin S., Zotti G., Ratcliffe C.I., Klug D.D., Gao X., Gao F. (2007). Synthesis and Thermoelectric Properties of Polycarbazole, Polyindolocarbazole, and Polydiindolocarbazole Derivatives. Chem. Mater..

[34] Han Z., Fina A. (2011). Thermal conductivity of carbon nanotubes and their polymer nanocomposites: A review. Prog. Polym. Sci..

[35] Yu C., Kim Y.S., Kim D., Grunlan J.C. (2008). Thermoelectric Behavior of Segregated-Network Polymer Nanocomposites. Nano Lett..

[36] Hiroshige Y., Ookawa M., Toshima N. (2006). High thermoelectric performance of poly(2,5-dimethoxyphenylenevinylene) and its derivatives. Synth. Met..

[37] Wakim S., Aïch B.R., Tao Y., Leclerc M. (2008). Charge transport, photovoltaic, and thermoelectric properties of poly(2,7-carbazole) and poly(indolo 3,2-b carbazole) derivatives. Polym. Rev..

[38] Liu H., Wang J., Hu X., Boughton R.I., Zhao S., Li Q., Jiang M. (2002). Structure and electronic transport properties of polyaniline=NaFe_4_P_12_ composite. Chem. Phys. Lett..

[39] Du Y., Shen S.Z., Cai K., Casey P.S. (2012). Research progress on polymer–inorganic thermoelectric nanocomposite materials. Prog. Polym. Sci..

[40] Sun Y., Wei Z., Xu W., Zhu D. (2010). A three-in-one improvement in thermoelectric properties of polyaniline brought by nanostructures. Synth. Met..

[41] Li J., Tang X., Li H., Yan Y., Zhang Q. (2010). Synthesis and thermoelectric properties of hydrochloric acid-doped polyaniline. Synth. Met..

[42] Yakuphanoglu F., Şenkal B.F., Saraç A. (2008). Electrical conductivity, thermoelectric power, and optical properties of organosoluble polyaniline organic semiconductor. J. Electron. Mater..

[43] Toshima N. (2002). Conductive polymers as a new type of thermoelectric material. Macromol. Symp..

[44] Yoon C.O., Reghu M., Moses D., Cao Y., Heeger A.J. (1995). Thermoelectricpower of doped polyaniline near the metal-insulator-transition. Synth. Met..

[45] Wang Z.H., Scherr E.M., MacDiarmid A.G., Epstein A.J. (1992). Transport and EPR studies of polyaniline-a quasi-one-dimensional conductor with 3-dimensional metallic states. Phys. Rev. B.

[46] Park Y.W., Yoon C.O., Lee C.H., Shirakawa H., Suezaki Y., Akagi K. (1989). Conductivity and thermoelectric-power of the newly processed polyacetylene. Synth. Met..

[47] Kaneko H., Ishiguro T., Takahashi A., Tsukamoto J. (1993). Magnetoresistance and thermoelectric-power studies of metal-nonmetal transition in iodine-doped polyacetylene. Synth. Met..

[48] Pukacki W., Płocharski J., Roth S. (1994). Anisotropy of thermoelectricpower of stretch-oriented new polyacetylene. Synth. Met..

[49] Park E.B., Yoo J.S., Choi H.J., Park J.Y., Park Y.W., Akagi K., Shirakawa H. (1995). Positive thermoelectric-power of alkali-metal-doped polyacetylene. Synth. Met..

[50] Yoon C.O., Na B.C., Park Y.W., Shirakawa H., Akagi K. (1991). Thermoelectricpower and conductivity of the stretch-oriented polyacetylene film doped with MOCl_5_. Synth. Met..

[51] Choi E.S., Suh D.S., Kim G.T., Kim D.C., Park Y.W. (1999). Magneto thermoelectric power of the doped polyacetylene. Synth. Met..

[52] Gao X., Uehara K., Klug D.D., Patchkovskii S., John S.T., Tritt T.M. (2005). Theoretical studies on the thermopower of semiconductors and low-band-gap crystalline polymers. Phys. Rev. B.

[53] Hiraishi K., Masuhara A., Nakanishi H., Oikawa H., Shinohara Y. (2009). Evaluation of thermoelectric properties of polythiophene films synthesized by electrolytic polymerization. Jpn. J. Appl. Phys..

[54] Yue R., Chen S., Lu B., Liu C., Xu J. (2011). Facile electrosynthesis and thermoelectric performance of electroactive free-standing polythieno[3,2-b]thiophene films. J. Solid State Electrochem..

[55] Bao L., Cong L., Shan L., Jing X., Feng J., Yu L., Zhuo Z. (2010). Thermoelectric performances of free-standing polythiophene and poly(3-methylthiophene) nanofilms. Chin. Phys. Lett..

[56] Kemp N.T., Kaiser A.B., Trodahl H.J., Chapman B., Buckley R.G., Partridge A.C., Foot P.J. (2006). Effect of ammonia on the temperature-dependent conductivity and thermopower of polypyrrole. J. Polym. Sci. B Polym. Phys..

[57] Aïch R.B., Blouin N., Bouchard A., Leclerc M. (2009). Electrical and Thermoelectric Properties of Poly(2,7-Carbazole) Derivatives. Chem. Mater..

[58] Chang K.C., Jeng M.S., Yang C.C., Chou Y.W., Wu S.K., Thomas M.A., Peng Y.C. (2009). The Thermoelectric Performance of Poly(3,4-ethylenedi oxythiophene)/Poly(4-styrenesulfonate) Thin Films. J. Electron. Mater..

[59] Scholdt M., Do H., Lang J., Gall A., Colsmann A., Lemmer U., Koenig J.D., Winkler M., Boettner H. (2010). Organic Semiconductors for Thermoelectric Applications. J. Electron. Mater..

[60] Feng J., Jing X., Bao L., Yu X., Rong H., Lai L. (2008). Thermoelectric performance of poly(3,4-ethylenedioxythiophene): Poly(styrenesulfonate). Chin. Phys. Lett..

[61] Moses D., Chen J., Denenstein A., Kaveh M., Chung T.C., Heeger A.J., MacDiarmid A.G., Park Y.W. (1981). Inter-Soliton Electron Hopping Transport in Trans-(CH)x. Solid State Commun..

[62] Zuzok R., Kaiser A.B., Pukacki W., Roth S. (1991). Thermoelectric power and conductivity of iodine-doped "new" polyacetylene. J. Chem. Phys..

[63] Park Y.W., Yoon C.O., Na B.C., Shirakawa H., Akagi K. (1991). Metallic Properties of Transition Metal Halides Dped Polyacetylene: The Solution Liquid State. Synth. Met..

[64] Pukacki W. (1994). Anisotropy of thermoelectric power of stretch-oriented new polyacetylene. Synth. Met..

[65] Yoon C.O., Reghu M., Moses D., Heeger A.J., Cao Y., Chen T.A., Wu X., Rieke R.D. (1995). Hopping transport in doped conducting polymers in the insulating regime near the metal-insulator boundary: Polypyrrole, polyaniline and poly alkylthiophenes. Synth. Met..

[66] Dubey N., Leclerc M. (2011). Conducting Polymers: Efficient Thermoelectric Materials. J. Polym. Sci. Part B Polym. Phys..

[67] Wu J., Sun Y., Xu W., Zhang Q. (2014). Investigating thermoelectric properties of doped polyaniline nanowires. Synth. Met..

[68] Wu J., Sun Y., Pei W.B., Huang L., Xu W., Zhang Q. (2014). Polypyrrole nanotube film for flexible thermoelectric application. Synth. Met..

[69] Liang L., Chen G., Guo C.Y. (2017). Polypyrrole nanostructures and their thermoelectric performance. Mater. Chem. Front..

[70] Kim D., Kim Y., Choi K., Grunlan J.C., Yu C. (2010). Improved Thermoelectric Behavior of Nanotube-Filled Polymer Composites with Poly(3,4-ethylenedioxythiophene) Poly(styrenesulfonate). ACS Nano.

[71] Kim G.H., Shao L., Zhang K., Pipe K.P. (2013). Engineered doping of organic semiconductors for enhanced thermoelectric efficiency. Nat. Mater..

[72] Culebras M., Gómez C.M., Cantarero A. (2014). Enhanced thermoelectric performance of PEDOT with different counter-ions optimized by chemical reduction. J. Mater. Chem. A.

[73] Jonas F., Heywang G., Werner S. (1988). Novel Polythiophenes, Process for Their Preparation, and Their Use. Bayer AG German Patent.

[74] Kim J.Y., Kwon M.H., Min Y.K., Kwon S., Ihm D.W. (2007). Self-Assembly and Crystalline Growth of Poly(3,4-ethylenedioxythiophene) Nanofilms. Adv. Mater..

[75] Li H., DeCoster M.E., Ireland R.M., Song J., Hopkins P.E., Katz H.E. (2017). Modification of the Poly(bisdodecylquaterthiophene) Structure for High and Predominantly Nonionic Conductivity with Matched Dopants. J. Am. Chem. Soc..

[76] Boudreault P.L., Blouin N., Leclerc M., Ullrich S., Dieter N. (2008). Poly(2,7-carbazole)s and related polymers. Polyfluorenes.

[77] Blouin N., Leclerc M. (2008). Poly(2,7-carbazole)s: Structure-Property Relationships. Acc. Chem. Res..

[78] Yao H., Fan Z., Cheng H., Guan X., Wang C., Sun K., Ouyang J. (2018). Recent Development of Thermoelectric Polymers and Composites. Macromol. Rapid Commun..

[79] Menke T., Ray D., Meiss J., Leo K., Riede M. (2012). In-situ conductivity and Seebeck measurements of highly efficient n-dopants in fullerene C_60_. Appl. Phys. Lett..

[80] Shi K., Zhang F., Di C.A., Yan T.W., Zou Y., Zhou X., Zhu D., Wang J.Y., Pei J. (2015). Toward High Performance n-Type Thermoelectric Materials by Rational Modification of BDPPV Backbones. J. Am. Chem. Soc..

[81] Huang D., Yao H., Cui Y., Zou Y., Zhang F., Wang C., Shen H., Jin W., Zhu J., Diao Y. (2017). Conjugated-Backbone Effect of Organic Small Molecules for n-Type Thermoelectric Materials with ZT over 0.2. J. Am. Chem. Soc..

[82] Ma W., Shi K., Wu Y., Lu Z.Y., Liu H.Y., Wang J.Y., Pei J. (2016). Enhanced Molecular Packing of a Conjugated Polymer with High Organic Thermoelectric Power Factor. ACS Appl. Mater. Interfaces.

[83] Schlitz R.A., Brunetti F.G., Glaudell A.M., Miller P.L., Brady M.A., Takacs C.J., Hawker C.J., Chabinyc M.L. (2014). Solubility-Limited Extrinsic n-Type Doping of a High Electron Mobility Polymer for Thermoelectric Applications. Adv. Mater..

[84] Wang Z.H., Ichimura K., Dresselhaus M.S., Dresselhaus G., Lee W.T., Wang K.A., Eklund P.C. (1993). Electronic transport properties of KxC70 thin films. Phys. Rev. B.

[85] Wang S., Sun H., Ail U., Vagin M., Persson P.O., Andreasen J.W., Thiel W., Berggren M., Crispin X., Fazzi D. (2016). Thermoelectric Properties of Solution-Processed n-Doped Ladder-Type Conducting Polymers. Adv. Mater..

[86] Sun Y., Sheng P., Di C., Jiao F., Xu W., Qiu D., Zhu D. (2012). Organic Thermoelectric Materials and Devices Based on p- and n-Type Poly(metal 1,1,2,2-ethenetetrathiolate)s. Adv. Mater..

[87] Sun Y., Qiu L., Tang L., Geng H., Wang H., Zhang F., Huang D., Xu W., Yue P., Guan Y.S. (2016). Flexible n-Type High-Performance Thermoelectric Thin Films of Poly(nickel-ethylenetetrathiolate) Prepared by an Electrochemical Method. Adv. Mater..

[88] Huang X., Sheng P., Tu Z., Zhang F., Wang J., Geng H., Zou Y., Di C.A., Yi Y., Sun Y. (2015). A two-dimensional pi-d conjugated coordination polymer with extremely high electrical conductivity and ambipolar transport behaviour. Nat. Commun..

[89] Xuan Y., Liu X., Desbief S., Leclère P., Fahlman M., Lazzaroni R., Berggren M., Cornil J., Emin D., Crispin X. (2010). Thermoelectric properties of conducting polymers: The case of poly(3-hexylthiophene). Phys. Rev. B.

[90] Schmidt R., Oh J.H., Sun Y.S., Deppisch M., Krause A.M., Radacki K., Braunschweig H., Könemann M., Erk P., Bao Z. (2009). High-Performance Air-Stable n-Channel Organic Thin Film Transistors Based on Halogenated Perylene Bisimide Semiconductors. J. Am. Chem. Soc..

[91] Nollau A., Pfeiffer M., Fritz T., Leo K. (2000). Controlled n-type doping of a molecular organic semiconductor: Naphthalenetetracarboxylic dianhydride (NTCDA) doped with bis(ethylenedithio)-tetrathiafulvalene (BEDT-TTF). J. Appl. Phys..

[92] Fukutomi Y., Nakano M., Hu J.Y., Osaka I., Takimiya K. (2013). Naphthodithiophenediimide (NDTI): Synthesis, structure, and applications. J. Am. Chem. Soc..

[93] Banal J.L., Subbiah J., Graham H., Lee J.K., Ghiggino K.P., Wong W.W. (2013). Electron deficient conjugated polymers based on benzotriazole. Polym. Chem..

[94] Takimiya K., Nakano M. (2018). Thiophene-Fused Naphthalene Diimides: New Building Blocks for Electron Deficient pi-Functional Materials. Bull. Chem. Soc. Jpn..

[95] Gross Y.M., Trefz D., Tkachov R., Untilova V., Brinkmann M., Schulz G.L., Ludwigs S. (2017). Tuning Aggregation by Regioregularity for High-Performance n-Type P(NDI2OD-T-2) Donor-Acceptor Copolymers. Macromolecules.

[96] Yang C.Y., Jin W.L., Wang J., Ding Y.F., Nong S., Shi K., Lu Y., Dai Y.Z., Zhuang F.D., Lei T. (2018). Enhancing the n-Type Conductivity and Thermoelectric Performance of Donor-Acceptor Copolymers through Donor Engineering. Adv. Mater..

[97] Chang M., Lim G., Park B., Reichmanis E. (2017). Control of Molecular Ordering, Alignment, and Charge Transport in Solution-Processed Conjugated Polymer Thin Films. Polym. Adv. Technol..

[98] Saglio B., Mura M., Massetti M., Scuratti F., Beretta D., Jiao X., McNeill C.R., Sommer M., Famulari A., Lanzani G. (2018). N-Alkyl substituted 1H-benzimidazoles as improved n-type dopants for a naphthalene-diimide based copolymer. J. Mater. Chem. A.

[99] Tietze M.L., Benduhn J., Pahner P., Nell B., Schwarze M., Kleemann H., Krammer M., Zojer K., Vandewal K., Leo K. (2018). Elementary steps in electrical doping of organic semiconductors. Nat. Commun..

[100] Liu J., Ye G., Zee B.V., Dong J., Qiu X., Liu Y., Portale G., Chiechi R.C., Koster L.J. (2018). N-Type Organic Thermoelectrics of Donor-Acceptor Copolymers: Improved Power Factor by Molecular Tailoring of the Density of States. Adv. Mater..

[101] Liu J., Qiu L., Alessandri R., Qiu X., Portale G., Dong J., Talsma W., Ye G., Sengrian A.A., Souza P.C. (2018). Enhancing Molecular n-Type Doping of Donor-Acceptor Copolymers by Tailoring Side Chains. Adv. Mater..

[102] Wang Y., Nakano M., Michinobu T., Kiyota Y., Mori T., Takimiya K. (2017). Naphthodithiophenediimide-Benzobisthiadiazole-Based Polymers: Versatile n-Type Materials for Field-Effect Transistors and Thermoelectric Devices. Macromolecules.

[103] Qiu L., Liu J., Alessandri R., Qiu X., Koopmans M., Havenith R.W., Marrink S.J., Chiechi R.C., Koster L.J., Hummelen J.C. (2017). Enhancing doping efficiency by improving host-dopant miscibility for fullerene-based n-type thermoelectrics. J. Mater. Chem. A.

[104] Shi W., Wu G., Hippalgaonkar K., Wang J.S., Xu J., Yang S.W. (2018). Poly(nickel-ethylenetetrathiolate) and Its Analogs: Theoretical Prediction of High-Performance Doping-Free Thermoelectric Polymers. J. Am. Chem. Soc..

[105] Kamarudin M.A., Sahamir S.R., Datta R.S., Long B.D., Sabri M., Faizul M., Mohd Said S. (2013). A review on the fabrication of polymer-based thermoelectric materials and fabrication methods. Sci. World J..

[106] Endo M., Hayashi T., Kim Y.A., Muramatsu H. (2006). Development and Application of Carbon Nanotubes. Jpn. J. Appl. Phys..

[107] He M., Qiu F., Lin Z. (2013). Towards high-performance polymer-based thermoelectric materials. Energy Environ. Sci..

